# Mineralocorticoid receptor antagonist use after hospitalization of patients with heart failure and post-discharge outcomes: a single-center retrospective cohort study

**DOI:** 10.1186/s12872-019-1175-3

**Published:** 2019-08-09

**Authors:** Matthew S. Durstenfeld, Stuart D. Katz, Hannah Park, Saul Blecker

**Affiliations:** 1 Department of Medicine, Division of Cardiology, University of California, San Francisco, Box 0124, C/O Salina Gu, San Francisco, CA 94143 USA; 2 Department of Medicine, New York University School of Medicine, 227 East 30th Street, New York, NY 10016 USA; 3 Department of Population Health, New York University School of Medicine, 227 East 30th Street, New York, NY 10016 USA

**Keywords:** Mineralocorticoid, Aldosterone, Heart Failure, Hospitalization

## Abstract

**Background:**

Mineralocorticoid receptor antagonists (MRA) are an underutilized therapy for heart failure with a reduced ejection fraction (HFrEF), but the current impact of hospitalization on MRA use is not well characterized. The objective of this study was to describe contemporary MRA prescription for heart failure patients before and after the full scope of hospitalizations and the association between MRA discharge prescription and post-hospitalization outcomes.

**Methods:**

We conducted a retrospective cohort study at an academic hospital system in 2013–2016. Among 1500 included hospitalizations of 1009 unique patients with HFrEF and without MRA contraindication, the mean age was 71.9 ± 13.6 years and 443 (29.5%) were female. We compared MRA prescription before and after hospitalizations with McNemar’s test and between patients with principal and secondary diagnoses of HFrEF with the chi-square test, and association of MRA discharge prescription with 30-day and 180-day mortality and readmissions using generalized estimating equations.

**Results:**

MRA prescriptions increased from 303 (20.2%) to 375 (25.0%) at discharge (+4.8%, *p* < 0.0001). More patients with principal diagnosis of HFrEF compared to those hospitalized for other reasons received MRA (34.9% versus 21.3%, *p* < 0.0001) and had them initiated (21.8% versus 9.7%, *p* < 0.0001). MRA prescription at discharge was not associated with mortality or readmission at 30 and 180 days, and there was no interaction with principal/secondary diagnosis.

**Conclusions:**

Among hospitalized HFrEF patients, 75% did not receive MRA before or after hospitalization, and nearly 90% of eligible patients did not have MRA initiated. As we found no signal for short-term harm after discharge, hospitalization may represent an opportunity to initiate guideline-directed heart failure therapy.

## Background

Heart failure is a major cause of hospital admission, particularly among older patients. By 2030, over 8 million Americans are projected to have heart failure with an estimated cost of $69.8 billion, with up to 80% of costs related to hospitalization [1]. High-quality trials have demonstrated that mineralocorticoid receptor antagonists (MRA) including spironolactone and eplerenone reduce mortality and readmissions among patients with heart failure with a reduced ejection fraction (HFrEF) compared to placebo [2–5]. In a meta-analysis, HFrEF patients treated with MRA had reduced mortality (Odds Ratio (OR) 0.77, 95% Confidence Interval (CI) 0.61–0.89) and reduced cardiovascular-specific hospitalization (OR 0.66, 95% CI 0.51–0.85) [6]. Based on the evidence, the American College of Cardiology/American Heart Association Guideline for the Management of Heart Failure includes a strong recommendation (Class IA) to use MRA in patients with left ventricular ejection fraction (LVEF) of 35% or less with New York Heart Association (NYHA) Class II-IV symptoms, estimated glomerular filtration rate (eGFR) >30 ml/min/1.73 m^2^, and serum potassium <5.0 mEq/L [7, 8].

Despite a strong level of evidence and inclusion in guidelines, MRA remain underutilized. MRA prescription rates for HFrEF patients range from 15–29% in the ambulatory setting [9–12] to 27–33% after heart failure hospitalization [11, 13–15]. Only 36% of veterans hospitalized with HFrEF in 2003–2009 who were “ideal candidates” for MRA received them, with significant practice variation and decrease in use over time to 31% by 2009 [16]. In a national sample of over 200,000 patients with HFrEF from 2009 to 2012, only 9% filled prescriptions for MRA [17].

Hospitalization may present an opportunity to increase adherence to guideline directed therapy, especially for patients with a secondary diagnosis of HFrEF. Data from almost a decade ago suggest that hospitalization, including heart-failure specific hospitalization, does not impact prescription of MRA [17]. Furthermore, patients with heart failure hospitalized for other reasons are less likely to receive guideline-directed medical therapy including MRA than patients hospitalized for heart failure [18]. Accordingly, the current study was conducted to describe contemporary MRA prescription for heart failure patients before and after the full scope of hospitalizations and the association between MRA discharge prescription and outcomes.

## Methods

We performed a retrospective cohort study of adults hospitalized within an academic tertiary-care hospital system from January 2013 to May 2016 with a principal or secondary discharge diagnosis of heart failure as defined by standard ICD-9CM and ICD-10 codes. Prior research suggests that the specificity of principal diagnosis of heart failure for acute decompensated heart failure is >95% with >87% positive predictive value for acute decompensated heart failure [19], and hospitalizations of patients hospitalized for other issues were coded with heart failure as a secondary diagnosis. We included patients with LVEF ≤35%, eGFR >30 ml/min/1.73 m^2^, serum potassium <5.0 mEq/L, and systolic blood pressure (SBP) ≥100 mmHg prior to discharge [7]. We excluded patients who died during the index admission, were discharged to hospice, or were pregnant. We identified our cohort using clinical data from the electronic health record (Epic Systems Corporation, Verona, Wisconsin). We extracted demographic, clinical, and billing information including MRA prescription before admission and at discharge and validated the data extraction with manual chart review of a subset of the cohort. To identify readmissions, patient data were linked to the New York State Planning and Research Cooperative System data registry, which includes all acute-care hospitalizations within New York State excluding federal hospitals such as Veterans Affairs hospitals [20]. To identify mortality events, patient data were linked to the New York State Vital Statistics registry. This study was approved by the New York University School of Medicine Institutional Review Board, and a waiver of consent was granted.

Our primary outcome was MRA prescription at the time of hospital discharge. We compared MRA prescription before and after hospitalization including change in MRA prescription status, MRA discontinuation, MRA prescription at discharge, and new MRA initiation with pre-specified principal and secondary diagnosis subgroups. Then we examined the association between MRA prescription at discharge and 30-day and 180-day outcomes of all-cause readmissions, heart-failure readmissions, hyperkalemia readmissions, and mortality. We defined heart failure readmissions by ICD-9-CM or ICD-10 codes for heart failure in the primary position and hyperkalemia-specific readmission by ICD-9-CM or ICD-10 hyperkalemia code in any position.

Patient demographic variables included age, sex, and race/ethnicity. Clinical data included LVEF, as measured during the hospitalization or within the preceding three months and extracted from echocardiogram reports as a structured data element, and first available systolic blood pressure from the visit. Laboratory values including sodium, potassium, and creatinine were collected at admission and the last values prior to discharge. Estimated glomerular filtration rates (eGFR) were calculated using the CKD-EPI formula [21]. Comorbid conditions including hypertension, atrial fibrillation/flutter, diabetes mellitus, chronic kidney disease, malignancy, cerebrovascular disease, peripheral vascular disease, acute myocardial infarction, chronic obstructive pulmonary disease, dementia, and cirrhosis were assigned based on discharge ICD-9-CM and ICD-10 codes. We recorded pre-admission and discharge medication prescriptions for the angiotensin converting enzyme (ACE)-inhibitors, angiotensin receptor blockers (ARB), beta-blockers, loop diuretics, and mineralocorticoid receptor antagonists.

### Statistical Analysis

We used McNemar’s test to compare MRA prescription before and after hospitalization in the entire cohort and with principal and secondary diagnosis subgroups. We used the chi-square test to compare discharge MRA prescriptions and new MRA initiation between principal and secondary diagnosis subgroups. To compare covariates among patients who did or did not receive MRA at discharge, we used the chi-square test for categorical variables and the t-test for continuous variables. Tests were evaluated at a two-sided significance level of *p* < 0.05.

We reported unadjusted readmissions and mortality at 30 days and 180 days for patients who were prescribed MRA versus those not prescribed MRA, with readmissions stratified into all-cause, heart failure principal diagnosis, and hyperkalemia-related. To compare adjusted mortality and readmissions at 30 days and 180 days for patients prescribed MRA therapy versus those not prescribed MRA, we developed models using generalized-estimating equations (GEE). The primary independent variable was MRA prescription at discharge. Readmissions were stratified into all-cause, heart failure principal diagnosis, and hyperkalemia-related. Because our study was observational rather than a randomized clinical trial, we adjusted the odds ratios based on factors that known to be associated with worse prognosis in heart failure. Our adjusted GEE models account for repeat hospitalizations of the same patient with adjustment for demographics, comorbid conditions, admission systolic blood pressure, admission sodium, discharge eGFR, and LVEF. We considered p-values <0.05 to be significant a priori and reported unadjusted and adjusted odds ratios with 95% confidence intervals.

To determine if there was a difference in outcomes with MRA prescriptions between patients with a principal and secondary diagnosis, we developed a second set of GEEs with an interaction term for discharge prescription and an indicator for heart failure diagnosis position as principal versus secondary. We adjusted these models for the same variables as the prior models. We considered p-values <0.10 to be significant for interaction a priori. Analyses were performed with STATA version SE 13.1 (StataCorp, College Station, TX).

## Results

Study population was represented by 1009 unique patients affected by heart failure, with 1500 hospitalizations (Table 1). The mean age of included patients was 71.9 ± 13.6 years and 443 (29.5%) were female (Table 1). Of these, 227 (15.1%) identified as black and 97 (6.5%) as Hispanic. Comorbid conditions were prevalent among our cohort: 76.7% had hypertension, 52.8% had atrial fibrillation, 41.1% had diabetes, and 34.1% had chronic kidney disease stages 1–3 (Table 1). Most patients were prescribed ACE inhibitors or ARB (59% before, 65% after), beta blockers (73% before, 78% after), and loop diuretics (58% before, 68% after) (Table 1). There were significant differences in comorbidities among patients with a principal as compared to secondary diagnosis of heart failure, including higher rates of diabetes and chronic kidney disease and lower rates of myocardial infarction (Table 1).

**Table 1:** Patient Characteristics by Principal vs Secondary Diagnosis (*N* = 1500)

	Total *N* = 1500	Principal HF Hospitalization *N* = 407	Secondary HF Hospitalization *N* = 1093	p-value
Demographics				
Age, Mean ± SD	71.9 ± 13.6	71.8 ± 13.6	71.9 ± 13.6	0.88
Female, n (%)	443 (29.5%)	110 (27.0%)	333 (30.5%)	0.19
Black	227 (15.1%)	87 (21.4%)	140 (12.8%)	<0.0001
Hispanic	97 (6.5%)	30 (7.4%)	67 (6.1%)	0.38
Comorbid conditions				
Hypertension	1151 (76.7%)	331 (81.3%)	820 (75.0%)	0.01
Atrial fibrillation	792 (52.8%)	211 (51.8%)	581 (53.2%)	0.65
Diabetes mellitus	617 (41.1%)	194 (47.7%)	423 (38.7%)	0.002
Chronic kidney disease	511 (34.1%)	164 (40.3%)	347 (31.8%)	0.002
Malignancy	325 (21.7%)	95 (23.3%)	230 (21.4%)	0.34
COPD	244 (16.3%)	81 (19.9%)	163 (14.9%)	0.02
Myocardial infarction	241 (16.1%)	41 (10.1%)	200 (18.3%)	0.0001
Cerebrovascular disease	204 (13.6%)	57 (14.0%)	147 (13.5%)	0.78
Peripheral vascular disease	170 (11.3%)	32 (7.9%)	138 (12.6%)	0.01
Dementia	141 (9.4%)	47 (11.6%)	94 (8.6%)	0.08
Cirrhosis	27 (1.8%)	7 (1.7%)	20 (1.8%)	0.89
LVEF %, Mean ± SD	26.4 ± 7.2	24.4 ± 7.5	27.2 ± 7.0	<0.0001
Admission Blood Pressure, Labs, & Home Medications				
SBP, Mean ± SD, mm Hg	130.7 ± 23.8	134.3 *±* 25.7	129.4 *±* 22.9	0.001
Sodium, Mean ± SD, mEq/L	137.4 ± 4.4	137.7 ± 4.4	137.4 ± 4.5	0.21
Potassium, Mean ± SD, mEq/L	4.4 ± 0.6	4.4 ± 0.6	4.4 ± 0.6	0.86
Creatinine, Mean ± SD, mg/dL	1.2 ± 0.5	1.3 ± 0.5	1.2 ± 0.5	0.11
eGFR, Mean ± SD, ml/min/1.73 m^2^	63.6 ± 24.9	61.4 ± 22.0	64.4 ± 25.9	0.03
ACE-inhibitor or ARB	882 (58.8%)	237 (58.2%)	635 (58.1%)	0.36
Beta-blocker	1095 (73.0%)	299 (73.5%)	796 (72.8%)	0.80
Loop diuretic	864 (57.6%)	275 (67.6%)	589 (53.9%)	<0.0001
Discharge Labs & Medications				
Creatinine, Mean ± SD, mg/dL	1.2 ± 0.4	1.2 ± 0.4	1.1 ± 0.4	<0.0001
eGFR, Mean ± SD, ml/min/1.73 m^2^	65.4 ± 24.0	61.4 ± 22.0	64.4 ± 25.9	<0.0001
Potassium, Mean ± SD, mEq/L	4.2 ± 0.4	4.2 ± 0.4	4.3 ± 0.4	0.01
ACE-inhibitor or ARB	975 (65.0%)	287 (70.5%)	688 (63.0%)	0.006
Beta-blocker	1184 (78.9%)	323 (79.4%)	861 (78.8%)	0.80
Loop diuretic	1022 (68.1%)	339 (83.3%)	683 (62.5%)	<0.0001
Primary Service*				
Cardiology	668 (44.5%)	284 (69.8%)	384 (35.1%)	<0.0001
Medicine	411 (27.4%)	85 (20.9%)	326 (29.8%)	0.0006
Other	395 (26.3%)	28 (6.9%)	367 (33.6%)	<0.0001

*Abbreviations*: *COPD* Chronic Obstructive Pulmonary Disease, *LVEF* left ventricular ejection fraction, *eGFR* estimated glomerular filtration rate (ml/min/1.73 m^2^), *ACE* angiotensin converting enzyme, *ARB* Angiotensin Receptor Blocker, *SBP* systolic blood pressure. *Primary Service totals do not add up to 100% due to missing data (~2% of admissions)

**Fig. 1:**
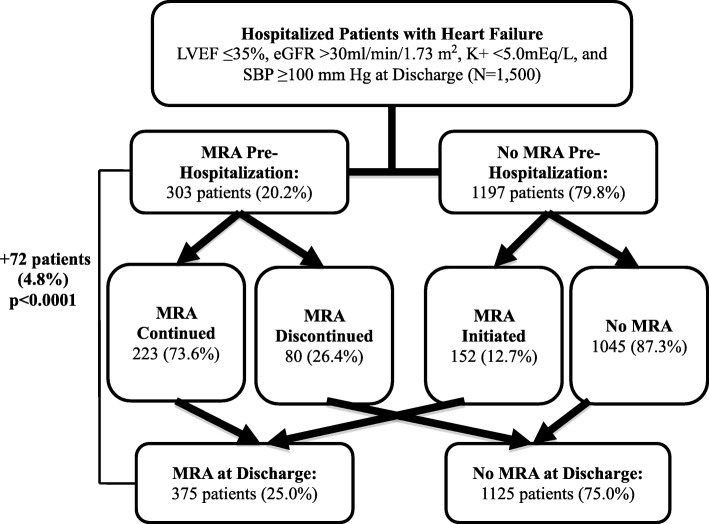
MRA Prescriptions Before and After Hospitalization (*N* = 1500). Change in MRA prescription before and after hospitalization (*N* = 1500). Overall, there was a net increase from 303 (20.2%) to 375 (25.0%) patients prescribed MRA at discharge compared to admission (*p* < 0.0001). Abbreviations: MRA = mineralocorticoid receptor antagonist, LVEF = left ventricular ejection fraction, eGFR = estimated glomerular filtration rate (ml/min/1.73 m^2^), K + =potassium (mEq/L), SBP = systolic blood pressure (mm Hg)

Among 1500 hospitalizations, 303 patients (20.2%) were prescribed MRA prior to hospitalization, and 375 patients (25.0%) were prescribed MRA at discharge for a net increase of 72 patients (+4.8%) prescribed MRA post-hospitalization (*p* < 0.0001, Figure 1). Of the 303 patients prescribed MRA prior to hospitalization, 223 patients (73.6%) continued and 80 patients (26.4%) discontinued MRA at discharge. Out of 1197 patients not prescribed MRA prior to hospitalization, 152 patients (12.7%) had MRA initiated and 1045 patients (87.3%) were never prescribed MRA. Patients who received MRA at discharge were younger (69.7 ± 13.6 versus 72.6 ± 13.2 years old, *p* = 0.0003), had lower LVEF (23.9 ± 7.0 versus 27.3 ± 7.1%, *p* < 0.0001), had lower admission systolic blood pressures (128.6 ± 23.3 versus 131.4 ± 23.9 mmHg, *p* = 0.0475), and were more likely to receive other guideline-directed medical therapies at discharge including ACE inhibitors or ARBs and beta blockers (Appendix).

Among 407 patients with a principal diagnosis of heart failure, 109 (26.8%) were prescribed MRA prior to hospitalization, and 142 (34.9%) were prescribed MRA at discharge for a net increase of 33 patients (+8.1%, *p* = 0.0008). Among 1093 patients with a secondary diagnosis of heart failure, 194 patients (17.7%) were prescribed MRA before hospitalization and 233 patients (21.3%) were prescribed MRA after hospitalization for a net increase of 3.6% (*p* = 0.0008). Patients hospitalized with a principal diagnosis of heart failure were more likely to receive MRA at discharge than patients hospitalized with a secondary diagnosis: 34.9% versus 21.3% (*p* < 0.0001). Likewise, patients with a principal diagnosis were also more likely to have MRA initiated: 21.8% versus 9.7% (*p* < 0.0001). There were no differences in MRA discontinuation among patients with a principal versus secondary diagnosis of heart failure: 29.4% versus 24.7% (*p* = 0.38).

Among 1500 hospitalizations, post-discharge outcomes were available for 1463. The 30-day mortality rate among patients prescribed MRA at discharge was 3.0% compared to 2.5% for patients not prescribed MRA at discharge (OR 1.23, 95% CI 0.61–2.51). The 180-day mortality rate was 9.3 and 10.2% among patients prescribed MRA and not prescribed MRA at discharge, respectively (OR 0.90, 95% CI 0.60–1.35). The 30-day all cause readmission rate was 22.2% for patients prescribed MRA and 21.6% for patients not prescribed MRA at discharge (OR 1.04, 95% CI 0.78–1.38). The 180-day all-cause readmission rate was 43.6% for patients prescribed MRA versus 46.4% for patients not prescribed MRA at discharge (OR 0.89, 95% CI 0.70–1.13). There were more 30-day heart failure specific readmissions among patients prescribed MRA than patients not prescribed MRA at discharge (7.4% versus 4.5%, OR 1.71, 95% CI 1.05–2.78). Similarly, patients prescribed MRA at discharge had more 180-day heart failure specific readmissions compared to those not prescribed MRA (20.8% versus 15.5%, OR 1.44, 95% CI 1.06–1.94). There were no differences in 30-day or 180-day hyperkalemia readmissions (Table 2).

**Table 2:** Post-Discharge Outcomes by Mineralocorticoid Receptor Antagonist (MRA) Prescription Status (*N* = 1463)

	MRA Prescribed at Discharge (*N* = 365)	MRA Not Prescribed at Discharge (*N* = 1098)	Unadjusted Odds Ratio for MRA Prescription (ref = no MRA) (95% CI)	Adjusted Odds Ratio for MRA Prescription (ref = no MRA) (95% CI)	Interaction between MRA and Principal/ Secondary Diagnosis, *p*-value
30-day Readmission	81 (22.2%)	237 (21.6%)	1.04 (0.78–1.38)	1.14 (0.84–1.57)	0.53
180-day Readmission	159 (43.6%)	509 (46.4%)	0.89 (0.70–1.13)	0.86 (0.67–1.11)	0.14
30-day Mortality	11 (3.0%)	27 (2.5%)	1.23 (0.61–2.51)	1.42 (0.67–3.03)	0.34
180-day Mortality	34 (9.3%)	112 (10.2%)	0.90 (0.60–1.35)	1.17 (0.76–1.79)	0.35
30-day Heart Failure Readmission	27 (7.4%)	49 (4.5%)	1.71 (1.05–2.78)	1.60 (0.95–2.68)	0.13
180-day Heart Failure Readmission	76 (20.8%)	170 (15.5%)	1.44 (1.06–1.94)	1.02 (0.72–1.44)	0.45
30-day Hyperkalemia Readmission	7 (1.9%)	13 (1.2%)	1.63 (0.65–4.12)	1.92 (0.70–5.24)	0.62
180-day Hyperkalemia Readmission	19 (5.2%)	52 (4.7%)	1.10 (0.64–1.89)	1.00 (0.55–1.84*)*	0.81

There were no associations between MRA prescription at discharge and short-term mortality and readmissions after adjusting for demographics, comorbid conditions, systolic blood pressure at admission, discharge estimated glomerular filtration rate, ejection fraction, and repeat hospitalizations of the same patient (Table 2). The associations between MRA prescription and heart failure specific readmissions at 30 days and 180 days were no longer statistically significant after adjustment, with adjusted odds ratios of 1.60 (95% CI 0.95–2.68) and 1.02 (95% CI 0.72–1.44), respectively. There were no statistically significant interactions between principal/secondary diagnosis status and MRA prescription at discharge with regards to all of the outcomes studied, with *p*-values for interaction ranging from 0.13 to 0.81 (Table 2).

## Discussion

Among hospitalized patients with heart failure with a reduced ejection fraction who met guideline-directed indications for MRA, use was infrequent both before and after hospitalization. We found a small increase in patients prescribed MRA from one out of five patients before hospitalization to one out of four patients after hospitalization. We found that MRA were underutilized due to a combination of ambulatory underuse preceding hospitalization (20%), high discontinuation rates during hospitalization (26%), and low initiation rates at discharge (13%). Our findings highlight an opportunity to identify appropriate candidates and initiate MRA throughout the continuum of care.

MRA utilization was particularly low for patients with HFrEF who were hospitalized for other causes: only 21% of patients with a secondary diagnosis of heart failure compared to 35% of patients with a principal diagnosis received MRA at discharge. Patients with a secondary diagnosis had lower rates of MRA prescription preceding hospitalization, lower rates of MRA initiation during hospitalization, and similar rates of discontinuation by time of discharge. It may be appropriate to have higher discontinuation rates and lower rates of MRA initiation for patients with diagnoses such as sepsis or gastrointestinal bleeding, but this does not explain lower rates of ambulatory prescription preceding hospitalization or similar rates of discontinuation that we found. Despite this caveat, these findings highlight a missed opportunity to initiate MRA, especially among patients with heart failure hospitalized for other reasons, which represent over three quarters of hospitalizations of patients with heart failure in the United States [22].

MRA prescription at discharge was not associated with differences in mortality and all-cause readmissions at 30 days and 180 days post-discharge, similar to a recent registry-based propensity-score matched cohort study [23]. In unadjusted analysis there were increased odds of heart failure specific readmission among patients who were versus who were not prescribed MRA at discharge; this difference was no longer significant after adjustment. Overall rates for hyperkalemia readmissions were low with no difference in rates among patients prescribed versus not prescribed MRA at discharge. Additionally, we found no differences in association of MRA use with outcomes for patients with a principal versus secondary diagnosis of heart failure. These findings, which should be considered exploratory analyses, suggest that prescribing MRA at discharge is not associated with adverse short-term outcomes and should be safe to initiate during care transitions.

Overall, our results that MRA are underutilized and frequently discontinued are consistent with prior studies, which predominantly included patients with acute decompensated heart failure [11, 13–15, 24–28]. Similar to our findings, one prior study that included patients with a secondary diagnosis of heart failure found that these patients were less likely to receive guideline-directed therapy including MRA than patients with a principal diagnosis of heart failure [18]. With significantly lower MRA prescription rates and MRA initiation rates among secondary heart failure patients, there is a greater opportunity to improve the quality of care for patients with a secondary diagnosis of HFrEF.

MRA are underused due to a combination of provider and patient-specific factors, primarily due to concerns about the risk of hyperkalemia [28]. Other barriers include knowledge gaps among physicians regarding patient eligibility, uncertainty regarding who should prescribe them during transitions of care, and concerns about polypharmacy, adverse effects, lack of follow-up, and non-adherence [9, 29]. To mitigate the risk of hyperkalemia, timely follow-up laboratory testing is critical especially in patients with underlying chronic kidney disease [30–32]. Inpatient MRA initiation increases appropriate laboratory follow-up to 25.2% from 2.8% for outpatient MRA initiation [33].

Despite these concerns, MRA prescription at discharge is associated with increased adherence and better outcomes. Patients are six times more likely to fill MRA prescriptions after heart failure hospitalization if they received an MRA prescription at discharge [11]. Likewise, among eligible patients not prescribed an MRA at discharge, only 5–13% subsequently had MRA initiated as outpatients [11, 27]. Increased MRA use in appropriate heart failure patients may be associated with decreased heart-failure readmissions [23, 26] and lower mortality [28, 34]. In summary, initiation of MRA for appropriate patients during hospitalization may increase the likelihood that patients receive MRA compared to deferring initiation to the outpatient setting, which may in turn improve outcomes.

### Limitations

First, as an observational study, the outcomes portion of our study may be subject to residual confounding and treatment selection bias. Second, by including a large number of patients with a secondary diagnosis of heart failure, we included some patients for whom it may not be appropriate to initiate an MRA at the time of discharge, such as patients admitted with sepsis or gastrointestinal bleeding. Third, we studied patients hospitalized within a single academic hospital system who may not be representative of the general population. Fourth, we did not determine whether patients filled or were adherent to MRA prescriptions provided on discharge, although prior data suggest that most patients prescribed MRA fill them [11]. Fifth, our study may have been underpowered for some 30-day outcomes including mortality and hyperkalemia readmissions, which we included as important safety outcomes. Sixth, our data lacked outpatient potassium results, so it is possible that we missed adverse events that did not require rehospitalization. Seventh, we did not collect data regarding use of device therapy such as implantable cardioverter-defibrillators and cardiac resynchronization therapy. This is important as both ICD and CRT may affect prognosis in patients with HFrEF, particularly those with diabetes, who may have higher burden of arrhythmias and differential responses to device therapy and interaction with MRA [35, 36]. Finally, NYHA class was not available and brain natriuretic peptide was not checked in the many patients, so it is possible that a small number of patients with a secondary diagnosis of heart failure had NYHA Class I symptoms, suggesting MRA therapy may not be strictly indicated for these patients according to current guidelines [7, 8, 26].

## Conclusions

MRA remain an under-utilized therapy in heart failure, and hospitalization is a missed opportunity to evaluate whether patients’ medications are optimized. Most hospitalized HFrEF patients who meet guideline-directed indication for MRA therapy do not receive MRA before or after hospitalization. Even fewer hospitalized heart failure patients have MRA newly initiated at discharge and many have them discontinued. The gap between current care and optimal guideline-directed medical therapy is even greater for patients with a secondary diagnosis of heart failure. Hospitalization appears to be a safe time to initiate MRA in appropriate patients to increase utilization of this evidence-based therapy without increased risks of harm.

## Appendix

**Table 3:** Patient characteristics by MRA discharge prescription

	Total *N* = 1500	MRA after Hospitalization *n* = 375	No MRA after Hospitalization *n* = 1125	*p*-value
Demographics				
Age, Mean ± SD	71.9 ± 13.6	69.7 ± 13.6	72.6 ± 13.2	0.0003
Female	443 (29.5%)	108 (28.8%)	335 (29.8%)	0.7193
Black	227 (15.1%)	67 (17.9%)	160 (14.2%)	0.0881
Hispanic	97 (6.5%)	37 (9.9%)	60 (5.3%)	0.0020
Comorbid conditions				
Hypertension	1151 (76.7%)	282 (75.2%)	869 (77.2%)	0.4171
Atrial fibrillation	792 (52.8%)	202 (53.9%)	590 (52.4%)	0.6328
Diabetes mellitus	617 (41.1%)	166 (44.3%)	451 (40.1%)	0.1545
Chronic kidney disease	511 (34.1%)	117 (31.2%)	394 (35.0%)	0.1762
Malignancy	325 (21.7%)	65 (17.3%)	260 (23.1%)	0.0187
COPD	244 (16.3%)	57 (15.2%)	187 (16.6%)	0.5181
Myocardial infarction	241 (16.1%)	61 (16.3%)	180 (16.0%)	0.9031
Cerebrovascular disease	204 (13.6%)	39 (10.4%)	165 (14.7%)	0.0369
Peripheral vascular disease	170 (11.3%)	37 (9.9%)	133 (11.8%)	0.3009
Dementia	141 (9.4%)	30 (8.0%)	111 (9.9%)	0.2834
Cirrhosis	27 (1.8%)	4 (1.1%)	23 (2.0%)	0.2174
LVEF %, Mean ± SD	26.4 ± 7.2	23.9 ± 7.0	27.3 ± 7.1	<0.0001
Admission Blood Pressure, Labs, & Home Medications				
Admission Systolic blood pressure, Mean ± SD	130.7 ± 23.8	128.6 ± 23.3	131.4 ± 23.9	0.0475
Sodium, Mean ± SD	137.4 ± 4.4	137.6 ± 4.1	137.4 ± 4.6	0.3131
Potassium, Mean ± SD	4.4 ± 0.6	4.4 ± 0.6	4.4 ± 0.6	0.7813
Creatinine, Mean ± SD	1.2 ± 0.5	1.2 ± 0.5	1.2 ± 0.5	0.6580
eGFR, Mean ± SD	63.6 ± 24.9	64.6 ± 23.3	63.3 ± 25.4	0.3398
ACE Inhibitor or ARB	882 (58.8%)	248 (66.1%)	634 (56.4%)	0.0009
Beta blocker	1095 (73.0%)	298 (79.5%)	797 (70.8%)	0.0011
Loop diuretic	864 (57.6%)	262 (69.9%)	602 (53.5%)	<0.0001
Discharge Labs & Home Medications				
Creatinine, Mean ± SD	1.2 ± 0.4	1.2 ± 0.4	1.1 ± 0.4	0.0817
eGFR, Mean ± SD	65.4 ± 24.0	64.7 ± 23.6	65.6 ± 24.1	0.5497
Potassium, Mean ± SD	4.2 ± 0.4	4.2 ± 0.4	4.2 ± 0.4	0.9968
ACE Inhibitor or ARB	975 (65.0%)	279 (74.4%)	696 (61.9%)	<0.0001
Beta blocker	1184 (78.9%)	336 (89.6%)	848 (75.4%)	<0.0001
Loop diuretic	1022 (68.1%)	315 (84.0%)	707 (62.8%)	<0.0001
Primary Service*				
Cardiology	668 (44.5%)	203 (54.1%)	465 (41.3%)	<0.0001
Medicine	411 (27.4%)	82 (21.9%)	329 (29.2%)	0.006
Other	395 (26.3%)	86 (22.9%)	309 (27.5%)	0.08

*Abbreviations*: *SD* Standard Deviation, *COPD* Chronic Obstructive Pulmonary Disease, *ACE* angiotensin converting enzyme, *ARB* Angiotensin Receptor Blocker, *eGFR* estimated glomerular filtration rate (ml/min/1.73 m^2^), *LVEF* left ventricular ejection fraction. *Primary Service totals do not add up to 100% due to missing data (~2% of admissions).

## Abbreviations

ACE: Angiotensin converting enzyme
ARB: Angiotensin receptor blocker
CI: Confidence interval
eGFR: Estimated glomerular filtration rate
GEE: Generalized estimating equations
HFrEF: Heart failure with a reduced ejection fraction
LVEF: Left ventricular ejection fraction
MRA: Mineralocorticoid receptor antagonist
NYHA: New York Heart Association
OR: Odds ratio
SBP: Systolic blood pressure
